# Creatine and post-viral fatigue syndrome: an update

**DOI:** 10.1080/15502783.2025.2517278

**Published:** 2025-06-06

**Authors:** Sergej M. Ostojic, Daren G. Candow, Mark A. Tarnopolsky

**Affiliations:** a University of Agder, Department of Nutrition and Public Health, Kristiansand, Norway; b University of Novi Sad, Applied Bioenergetics Lab, Faculty of Sport and Physical Education, Novi Sad, Serbia; c University of Pécs, Faculty of Health Sciences, Pécs, Hungary; d University of Regina, Faculty of Kinesiology and Health Studies, Regina, SK, Canada; e Department of Pediatrics, McMasterChildren’s Hospital, Hamilton, ON, Canada

**Keywords:** Creatine monohydrate, bioenergetics, fatigue, brain metabolism, clinical symptoms

## Abstract

Post-viral fatigue syndrome, classified as a neurological condition by the WHO (ICD-11 code: 8E49), manifests as persistent fatigue, cognitive difficulties, and post-exertional malaise following viral infections. It shares commonalities with chronic fatigue syndrome and myalgic encephalomyelitis but is distinct due to its association with preceding viral events. Emerging research identifies bioenergetic disruptions, particularly mitochondrial dysfunction and impaired creatine metabolism, as key contributors. Recent studies suggest creatine supplementation may alleviate symptoms and improve energy metabolism. This narrative review summarizes recent advancements in utilizing creatine as a diagnostic and therapeutic target for post-viral fatigue syndrome and explores future directions for its application in managing this perplexing condition.

## 1. Fundamentals of post-viral fatigue syndrome

According to the 11^th^ Revision of the International Classification of Diseases, a comprehensive global health classification system developed by the World Health Organization, post-viral fatigue syndrome (PVFS) is a neurological condition characterized by prolonged and debilitating fatigue that persists following a viral infection. It is classified under “Other disorders of the nervous system,” and its specific code is 8E49 (https://icd.who.int/en). PVFS often includes symptoms such as cognitive difficulties (commonly referred to as “brain fog”), intermittent and unrefreshing sleep, and post-exertional malaise, which involves a worsening of symptoms after physical or mental exertion [1]. It shares considerable overlap with diagnoses such as chronic fatigue syndrome and myalgic encephalomyelitis, but it is distinct in its direct association with an antecedant viral infection. However, PVFS is often considered an umbrella term that encompasses these and other related conditions, including fibromyalgia, due to their commonalities in symptoms, prognosis, and management strategies [2,3]. The condition is often challenging to diagnose as there are no definitive diagnostic tests, and symptoms vary widely in severity among individuals [4]. Management typically focuses on symptom alleviation and support, with approaches like pacing activity levels to avoid symptom exacerbation. The prevalence of post-viral fatigue syndrome in the general population is not precisely defined and varies depending on the study and diagnostic criteria. Estimates suggest that a significant proportion of individuals experience prolonged fatigue after viral infections, with rates ranging from 10% to 35% following illnesses such as mononucleosis, COVID-19, or influenza [1]. However, the prevalence of formally diagnosed PVFS is much lower [5], as many cases go undiagnosed or overlap with other conditions. Post-viral fatigue syndrome after COVID-19 pandemics (also known as post-COVID-19 condition or long COVID) raised interest about this perplexing condition in terms of addressing diagnosis, prognosis, and management. Despite advancements, many questions regarding the therapeutics and diagnostics of PVFS remain unresolved [6], extending beyond the scope of this paper. Emerging evidence underscores the potential role of creatine, a naturally occurring amino acid derivative, as both a diagnostic and therapeutic target for PVFS. This narrative review serves as an update to a previous comprehensive review [7], examining imbalances in creatine metabolism associated with fatigue syndromes and exploring current and future research directions for its application in managing this complex condition.

## 2. PVFS etiology: the role of impaired bioenergetics

The etiology of PVFS is multifactorial and not fully elucidated. It likely involves a combination of immune dysregulation, persistent inflammation, and metabolic disturbances triggered by a viral infection [8]. Chronic activation of the immune system, mitochondrial dysfunction, and disruptions in the hypothalamic-pituitary-adrenal axis are frequently implicated [9]. Certain viruses, such as Epstein-Barr virus, influenza, or SARS-CoV-2 May 2001induce PVFS due to prolonged immune responses and/or inadequate resolution of inflammation [10]. Psychosocial factors and preexisting conditions may also contribute to individual susceptibility [11]. Beside other factors, energy imbalances appear to play a significant role in the etiology of PVFS. Disruptions in cellular energy metabolism, particularly within the mitochondria, are frequently observed in affected individuals [12]. Viral infections may impair mitochondrial function, leading to reduced production of adenosine triphosphate (ATP), increased oxidative stress, and an accumulation of metabolic byproducts that further exacerbate energy deficits. These dysfunctions contribute to the hallmark fatigue of PVFS, as the body struggles to meet energy demands. A hypometabolic state with impaired sphingolipid abundance and reduction in mitochondrial substrate oxidation may also contribute to the persistent energy imbalance observed in PVFS [13]. The metabolism of creatine, a critical molecule for ATP replenishment, appears to be also impaired in PVFS. While comprehensive information on creatine imbalances in PVFS has been previously documented [7], emerging studies emphasize the significance of creatine deficits in key organs affected by the condition. For instance, a recent study demonstrated significantly reduced creatine levels in the anterior cingulate cortex of 22 PVFS patients compared to 13 healthy controls [14]. Similarly, research identified substantial reductions in skeletal muscle and brain creatine levels in 19 individuals with post-COVID fatigue syndrome, with lower creatine levels correlating with increased symptom severity [15]. Further, an analysis of 81 patients recovering from severe COVID-19 infection confirmed significant cerebral creatine depletion, highlighting profound metabolic disruptions in PVFS [16]. Creatine reduction may be present in several groups of patients with post-COVID fatigue, potentially driven by excessive energy demands during recovery. Several studies have reported mild to moderate alterations in surrogate biomarkers of creatine metabolism in the circulation across diverse COVID-19 populations, including pediatric patients [17,18], individuals with poor clinical outcomes [19–21], and those with pre-existing comorbidities [22]. These observations highlight the potential significance of reduced brain and muscle creatine levels as both a metabolic indicator and a prospective therapeutic target in PVFS and related disorders.

Despite emerging interest, the assessment of biomarkers related to creatine imbalances has not yet been incorporated into routine diagnostic protocols for PVFS, likely due to several key factors. First, the underlying pathophysiology of PVFS remains incompletely understood, and the involvement of creatine metabolism has only recently been proposed as a potential contributing mechanism. Although initial findings suggest that disruptions in creatine homeostasis may be relevant to fatigue-related conditions, the current evidence base lacks the robustness and consistency necessary to justify clinical adoption. Second, there is no standardized or widely accepted panel of biomarkers specifically designed to detect creatine imbalances in PVFS. While creatine and its metabolites can be measured in biological fluids such as blood or urine, or through advanced techniques like proton magnetic resonance spectroscopy (^1^ H-MRS), these methods are often costly, technically complex, and may lack diagnostic specificity for this condition. Moreover, the translation of novel biomarkers into clinical practice requires extensive validation through large-scale, well-controlled studies, regulatory approval, and broad clinical consensus – processes that have not yet been fulfilled for creatine-related markers in PVFS. Continued research is essential to elucidate the mechanistic connections between creatine metabolism, energy dysregulation, and clinical symptoms in PVFS, which may ultimately inform the development of reliable diagnostic tools.

## 3. Creatine supplementation for post-viral fatigue syndrome

Management of PVFS, including post-COVID fatigue, primarily aims to alleviate symptoms and provide supportive care. Approaches often include cognitive behavioral therapy and graded exercise therapy to manage fatigue and improve function, and pharmacological treatments to address specific symptoms such as sleep disturbances, depression, or chronic pain [23,24]. Dietary interventions have also gained attention for their potential to support energy metabolism [25]. Among nutritional strategies, creatine has garnered scientific interest for its potential role in addressing PVFS and related conditions due to its impact on cellular energy dynamics [26,27]. Its use for managing PVFS was minimally explored prior to the COVID-19 pandemic, with no studies focusing exclusively on creatine supplementation for this condition. A broader examination of preliminary studies can be found elsewhere [7]. Interest in creatine increased significantly after the COVID pandemic, where it was proposed as a potential component of pulmonary rehabilitation protocols for COVID-19 patients [28]. This recommendation is based on evidence suggesting that creatine supplementation may offer benefits during the rehabilitation of individuals with various pulmonary conditions, such as chronic obstructive pulmonary disease, cystic fibrosis, and respiratory failure [29,30]. These benefits are thought to arise from creatine’s role in enhancing cellular energy metabolism, potentially improving muscle function, exercise capacity, and overall quality of life in affected patients. However, the literature presents a mixed picture, as other studies have reported minimal or no significant effects of creatine supplementation in similar patient populations [31,32]. These discrepancies may be attributed to differences in study design, sample size, supplementation protocols, or baseline nutritional and functional status of participants. Thus, while creatine holds promise as an adjunct therapy in pulmonary rehabilitation, further well-controlled, large-scale trials are needed to establish its efficacy and to identify which subgroups of patients are most likely to benefit.

Recent interventional studies have evaluated the effects of creatine supplementation for post-COVID fatigue syndrome. A six-month regimen of creatine monohydrate supplementation (4 g/day) demonstrated significant improvements in patient- and clinician-reported outcomes, as well as enhanced brain and muscle bioenergetics, in 12 patients [33]. Similar findings emerged from a three-month randomized controlled trial combining creatine (4 g/day) with breathing exercises, which reduced respiratory discomfort and improved cognitive function in 8 patients [34]. Additional studies showed creatine’s benefits on fatigue, cognition, and tissue bioenergetics, even in shorter trials [34], and across non-randomized designs [35] with daily dosages up to 16 grams. A summary of interventional studies with creatine and creatine analogs in post-viral fatigue syndrome and similar conditions are presented in Table 1. Overall, these findings highlight creatine’s potential to address the bioenergetic challenges seen in PVFS and related disorders. While promising, the studies underscore the need for larger, well-controlled trials to validate these findings and establish optimal dosing regimens, especially given the relatively modest increase in total creatine in most regions of the brain (<10%) in response to high daily creatine monohydrate intake (20 g/d) [42]. Creatine’s safety profile and accessibility make it a strong candidate for broader use, particularly for individuals with limited dietary creatine intake or those unable to engage in strenuous physical activity. As research progresses, integrating creatine into standard PVFS management protocols could address critical gaps in current care, offering a novel approach to mitigating the impacts of this debilitating condition. Interestingly, a recent study highlighted the potential of alternative a non-creatine based synbiotic mixture intervention to enhance low brain creatine levels in individuals with PVFS. The findings demonstrated that improvements in brain creatine concentrations were associated with enhanced clinical outcomes, suggesting a promising avenue for therapeutic strategies targeting creatine metabolism [43].

**Table 1.** Interventional studies with creatine and creatine analogs in post-viral fatigue syndrome and similar conditions.

Reference	Design	Condition	Intervention	*n*	Creatine dosage	Duration	Main effects
[36]	RCT	PVFS	CRM w polinutrients	53	3.0 g/d	10 wks	No effects
[37]	Case study	Fibromyalgia	CRM w psychotopics	1	3.0 g/d for 7 days 5.0 g/d afterwards	4 wks	Reduced depression Improved health-related quality of life Improved sleep and somatic symptoms
[38]	Open label	Fibromyalgia	CRM w existing therapy	30	3.0 g/d for 3 wks 5.0 g/d afterwards	8 wks	Mittigated disease scores Reduced pain Improved sleep and health-related quality of life
[39]	RCT	Fibromyalgia	CRM	43	20.0 g/d for 5 days 5.0 g/d afterwards	4 mos	Increased muscle creatine Increased dynamic and isometric strength No changes in cardiorespiratory endurance No changes in pain, quality of life, and cognition
[40]	RCT	PVFS	GAA	21	2.4 g/d	3 mos	Increased muscle creatine Increased quadriceps strength and aerobic power Improved health-related quality of life No changes in general fatigue and pain scores
[33]	RCT	PVFS	CRM	12	4.0 g/d	6 mos	Increased muscle and brain creatine Improved clinical features * No changes in fatigue and exercise tolerance
[34]	RCT	PVFS	CRM w breathing exercise	8	4.0 g/d	3 mos	Increased muscle and brain creatine Reduced post-exercise malaise No changes in fatigue and exercise tolerance
[41]	RCT	PVFS	CRM w/wo glucose	15	8.0 g/d	2 mos	Increased brain creatine No changes in clinical features No changes in fatigue and exercise tolerance
[35]	Open-label	PVFS	CRM	11	16.0 g/d	6 wks	Increased brain creatine Increased handgrip strength Reduced fatigue and reaction time

*Abbreviations*: RCT, randomized controlled trial; CRM, creatine monohydrate; GAA, guanidinoacetic acid (precursor of creatine). Asterisk (*) encompass following clinical features: anosmia, ageusia, breathing difficulties, lung pain, body aches, headaches, and difficulties concentrating.

## 4. Future frontiers

**Figure 1.**
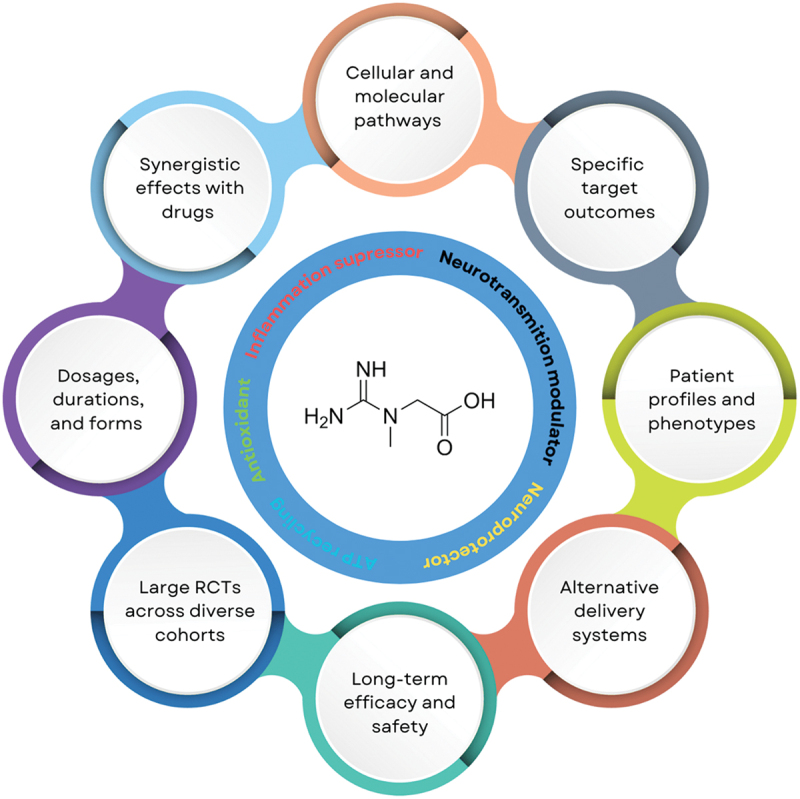
Emerging frontiers for the application of creatine in post-viral fatigue syndrome.

Although creatine levels and creatine supplementation show diagnostic and therapeutic potential, significant research is still required before they can be fully recognized as a part of routine clinical management of PVFS. Addressing several critical limitations observed in current clinical trials – namely, the small sample sizes of participants with PVFS, the inconsistent dosing regimens of creatine supplementation, and the narrow range of biomarkers employed to monitor therapeutic response – will be essential for informing and improving the design of future research in this domain. Small and often heterogeneous cohorts limit the generalizability and statistical power of existing findings, while variability in dosage and duration of creatine administration complicates the interpretation and comparison of outcomes across studies. Furthermore, reliance on a limited set of biomarkers restricts our understanding of the mechanistic pathways through which creatine may exert its effects. Future frontiers could thus include several areas of exploration, some depicted in Figure 1. First, more mechanistic studies are required to investigate the cellular and molecular pathways through which creatine influences energy metabolism, mitochondrial function, and oxidative stress in PVFS. It is also important to identify subgroups within PVFS patients who are most likely to benefit from creatine supplementation based on biomarkers, genetic profiles, or disease phenotypes. Additional research is warranted to explore different dosages, durations, and forms of creatine (*e.g*., monohydrate *vs*. other formulations), and/or multi-ingredient combinations (*e.g*., omega-3 fatty acids, carbohydrate, antioxidants, mitochondrial enhancers) tailored for PVFS management. Studying synergistic effects of creatine with other interventions, such as cognitive-behavioral therapy, sleep optimization, exercise protocols, or adjunctive nutraceuticals might be required to develop effective combination therapies. We need to identify specific target outcomes by assessing creatine’s impact on not only fatigue but also cognitive function, immune dysregulation, and mood disturbances commonly associated with PVFS. Examining the long-term safety and efficacy of creatine use in populations suffering from PVFS is another frontier, especially given the chronic nature of the syndrome, along with conducting larger, randomized controlled trials across diverse populations to confirm preliminary findings and establish standardized treatment protocols. Developing and rigorously evaluating novel and alternative delivery systems, such as creatine-enriched foods or transdermal applications, is essential to enhance accessibility and adherence, particularly in PVFS populations with unique needs or limitations. These directions aim to optimize the therapeutic potential of creatine, addressing gaps in current understanding and improving the quality of life for PVFS patients.

## 5. Conclusion

Post-viral fatigue syndrome represents a complex and multifactorial condition characterized by persistent fatigue, cognitive impairments, and bioenergetic disruptions following viral infections. Despite recent advancements, challenges persist in understanding its pathophysiology, identifying precise diagnostic markers, and developing effective therapies. Emerging evidence highlights the role of mitochondrial dysfunction and creatine metabolism in PVFS, positioning creatine as a promising therapeutic candidate. While initial studies suggest its efficacy in alleviating fatigue and improving bioenergetics, further large-scale, rigorous trials are essential. Addressing these gaps will pave the way for optimized care and improved patient outcomes.

## Data Availability

All data analyzed are included in the article. Further inquiries can be directed to the corresponding author.
